# Physical-Mechanical Properties and Micromorphology of Calcium Cements Exposed to Polyacrylic and Phosphoric Acids

**DOI:** 10.1155/2018/3197510

**Published:** 2018-05-09

**Authors:** Gustavo Fernandes de Souza, Ana Beatriz Arrais, Cícero Flávio Soares Aragão, Isana Alvares Ferreira, Boniek Castillo Dutra Borges

**Affiliations:** ^1^Department of Dentistry, Federal University of Rio Grande do Norte (UFRN), Natal, RN, Brazil; ^2^Department of Pharmacy, Federal University of Rio Grande do Norte (UFRN), Natal, RN, Brazil

## Abstract

**Objective:**

To evaluate if physical and mechanical properties of self-curing calcium hydroxide cements were affected by contact with polyacrylic and phosphoric acids.

**Materials and Methods:**

Resin-containing (Life (LF)) and resin-free (Hydro C (HyC)) materials were subjected to polyacrylic acid conditioning and rinsing (POL); phosphoric acid conditioning and rinsing (PHO); rinsing only; and no treatment (*n* = 10). Water sorption/solubility, release of hydroxyl ions (pH), roughness (Ra), and impact resistance were evaluated. Additional samples (*n* = 1) were prepared for scanning electron microscopy (SEM) analysis of the surface morphology. Data were analyzed by two-way ANOVA and Tukey post hoc test (*P* < 0.05).

**Results:**

Water sorption was significantly higher for LF when in contact with PHO and lower for POL (*P* < 0.05). The mean solubility was higher with POL for both cements (*P* < 0.05). PHO increased the mean surface roughness for HyC (*P* < 0.01); a significant decrease was noted for LF after contact with both acids (*P* < 0.01). PHO promoted lower release of hydroxyl ions on both cements (*P* < 0.05). For LF, rinsing, PHO, and POL presented similar morphology, differing from the control group. For HyC, PHO and POL presented similar morphology, differing from the control group.

**Conclusions:**

PHO had a negative effect on the physical properties of the cements tested, except for the solubility test. POL affected roughness and solubility of HyC cement.

**Clinical Relevance:**

Clinical procedures that require polyacrylic and phosphoric acid conditioning must be done carefully on self-curing calcium hydroxide cements in order to avoid negative impact on their properties.

## 1. Introduction

Calcium hydroxide cement is traditionally chosen for indirect pulp capping of deep cavities [1, 2]. In addition to low cost and ease of handling [3], self-curing calcium hydroxide cements (SCCHCs) also present the following properties: biocompatibility and alkalinity [4], ability to stimulate dentin formation [4, 5], antibacterial properties [5], thermal insulation [6], and protecting the pulp from any irritating components in restorative materials [1]. However, the physical and mechanical integrity of SCCHCs should not be affected by any further treatment performed during tooth restoration.

After indirect pulp capping with SCCHC, glass ionomer cement and/or an adhesive system are usually placed in the cavity before insertion of composite resin. Polyacrylic and phosphoric acid conditioning are recommended before the application of glass ionomer cement and etch-and-rinse adhesive systems, respectively [7, 8]. Because of the difficulty in restricting the acid application to dentin and/or enamel walls, polyacrylic and phosphoric acids may come in contact with the SCCHC, which might lead to alterations in the physical and mechanical properties of the SCCHC, despite the lack of scientific evidence on this subject.

Properties such as water sorption, solubility, and release of hydroxyl ions (pH) are related to the ability to maintain an alkaline state, which is necessary to regulate odontoblast mineralization [9], and impact resistance is detrimental to mechanical strength under compressive forces during tooth function [2, 10]. The surface roughness and morphology can indicate material degradation after exposure to external agents [11, 12]. However, there is no evidence that contact of SCCHCs containing different components with phosphoric/polyacrylic acids does not affect water sorption, solubility, release of hydroxyl ions (pH), surface roughness, impact resistance, and morphology. The efficacy of SCCHCs as pulp capping agents would be compromised if phosphoric and polyacrylic acids affect those properties.

This study aims to evaluate the water sorption, solubility, release of hydroxyl ions (pH), surface roughness, impact resistance, and morphology of SCCHCs after contact with polyacrylic and phosphoric acids. The null hypothesis tested is that the acids would have no influence on the physical and mechanical properties tested for both cements.

## 2. Methods and Materials

### 2.1. Experimental Design

This in vitro study involved a 2 × 4 factorial design. The factors under study were material (a resin-containing SCCHC (Life (LF)) and a resin-free SCCHC (Hydro C (HyC))) and conditioning agent (polyacrylic conditioning and rinsing (POL); phosphoric acid conditioning and rinsing (PHO); rinsing only; and no treatment). The response variables were water sorption, solubility, release of hydroxyl ions (pH), and surface roughness. A secondary response variable was surface morphology. The materials used in this study are described in Table 1.

### 2.2. Water Sorption and Solubility

Forty disk-shaped samples (1 mm thick × 5 mm diameter) of each SCCHC were prepared using polyvinyl siloxane prefabricated molds. Then, 0.2 mL of base and catalyst pastes was dispensed on a glass plate and mixed for 10 s with a spatula until the mixture was homogeneous, according to the manufacturer's instructions. The mixture was immediately placed into the mold, which was covered with a polyester strip and a glass plate until curing.

After curing, the samples were removed from the molds and subjected to the conditioning agents (*n* = 10) as follows:

- POL: 0.1 mL of polyacrylic acid was applied on the surface of the sample for 15 s, then rinsed with distilled water (20 mL) for 15 s, and dried with oil-free air.
- PHO: 0.1 mL of phosphoric acid was applied on the surface of the samples for 15 s, then rinsed with distilled water (20 mL) for 15 s, and dried with oil-free air.
- Rinsing only: samples were rinsed with distilled water (20 mL) for 15 s.
- No treatment: samples were not subjected to any acid or rinsing.

Water sorption and solubility analysis was performed on the samples in compliance with ISO 4049 (except for the specimen dimensions). The samples were stored in desiccators containing silica gel at 37°C and weighed daily in an analytical balance (Tel Marke, Bel Quimis, São Paulo, SP, Brazil) accurate to 0.001 mg, constituting a weighing cycle every 24 h. The complete cycle was repeated until a constant mass (m1) was obtained. The thickness (four measurements at four equal points on the circumference) and diameter (two measurements at right angles) of each specimen were measured using a digital electronic caliper (Mitutoyo Corporation, Tokyo, Japan). Mean values were used to calculate the volume (V) of each specimen (in mm^3^). Samples were stored in 20 mL of distilled water per sample (pH 6.46) at 37°C for 7 days and weighed after being carefully wiped with absorbent paper to record m2. The specimens were then returned to the desiccators. The entire mass reconditioning cycle was repeated, and the constant mass was recorded as m3. The values for water sorption (WS) and solubility (WSB) (*μ*g/mm^3^) were calculated using the following equations:

(1) $$\begin{array}{c} WS=\frac{\left(m2-m3\right)}{V},\\ WSB=\frac{\left(m1-m3\right)}{V}. \end{array}$$

### 2.3. Roughness, Impact Resistance, and Release of Hydroxyl Ions (pH)

Forty disk-shaped samples (1 mm thick × 5 mm diameter) of each SCCHC were prepared and subjected to conditioning agents as described for the water sorption/solubility analyses. Samples were stored for 7 days in distilled water (20 mL, pH 6.46) at 37°C to reproduce the same storage period as for the analysis of water sorption/solubility.

Surface roughness was measured with a profilometer (Precision Surtronic 25, Taylor Hobson, UK) in *μ*m, which has a piezoelectric transducer. The measuring length of 1.25 mm and cut-off of 0.25 mm were used. Three measurements were made in the center of the samples, turning the specimen in 120°, approximately, and the arithmetic mean was obtained [13].

Immediately after the surface roughness measurements, impact resistance was evaluated using a crushing strength tester (298-ATTS, Nova Ética, Vargem Grande do Sul, SP, Brazil). Samples were positioned on the apparatus to receive diametrically applied pressure. The crushing strength was obtained in newtons.

The distilled water used to store the samples was collected for the pH analysis. The pH values were determined with using a digital pH meter (PHS-3E, Jiangshu Instruments, Jiangshu, China) previously calibrated at room temperature (25 ± 2°C) with standard buffers of pH 6.86 and 4.00.

### 2.4. Surface Morphology

Additional samples of each SCCHC were fabricated and subjected to the conditioning agents for evaluation of the surface morphology by scanning electron microscopy (SEM). Samples were mounted on aluminum stubs with sticky double-sided conductive metal tape without special treatment. A TM 3000 table-top SEM (Hitachi High-Technologies Corporation, Tokyo, Japan) was used at 10 kV accelerating voltage. Image acquisition was performed by TM 3000 microscope software, version 02-01 (Hitachi High-Technologies Corporation, Tokyo, Japan). Images were obtained immediately after each conditioning agent was applied and after storing in distilled water for 7 days at 37°C.

### 2.5. Statistical Analysis

Data were analyzed by two-way ANOVA and Tukey post hoc test (*P* < 0.05) using the ASSISTAT beta software (UFCG, Campina Grande, PB, Brazil).

## 3. Results

### 3.1. Water Sorption and Solubility

For water sorption, ANOVA showed that there were statistically significant differences in the interaction material versus conditioning agent (*P* < 0.05). Comparisons among the groups are listed in Table 2. For LF, POL and PHO had the lowest and highest means, respectively, although they provided similar means for the control and rinsing only. There was no statistically significant difference between conditioning agents for HyC. Comparing both materials for the same conditioning agent, only POL showed statistically significant differences; HyC had a higher mean than LF.

For solubility, ANOVA showed that there were statistically significant differences in the interaction material versus conditioning agent (*P* < 0.01). Comparisons among the groups are listed in Table 3. For HyC, POL and PHO had the highest and lowest means, respectively, although they provided similar means for no treatment and rinsing only. For LF, POL had a statistically higher mean than other conditioning agents. Comparing both materials for the same conditioning agent, there were no statistically significant differences between them.

### 3.2. Roughness

ANOVA showed that there were statistically significant differences among conditioning agents (*P* < 0.01), between materials (*P* < 0.01), and in the interaction material versus conditioning agent (*P* < 0.01). Comparisons among groups are listed in Table 4. For HyC, PHO had statistically higher means than other conditioning agents. For LF, POL and PHO had statistically lower means than rinsing only and no treatment. Comparing both materials for the same conditioning agent, LF had higher means than HyC for rinsing and no treatment. LF had a lower mean than HyC for PHO, and LF and HyC had similar means for POL.

### 3.3. Impact Resistance

ANOVA showed statistically significant differences between materials (*P* < 0.01). Comparisons among the groups are listed in Table 5. For both materials, there were no statistically significant differences among conditioning agents. Comparing both materials for the same conditioning agent, only POL had statistically significant differences between them; HyC had a higher mean than LF.

### 3.4. Release of Hydroxyl Ions (pH)

ANOVA showed that there were statistically significant differences in the interaction material versus conditioning agent (*P* < 0.05). Comparisons among groups are listed in Table 6. For both cements, PHO had statistically lower means than other conditioning agents. Comparing both materials for the same conditioning agent, only PHO had statistically significant differences between them; LF had higher mean than HyC.

### 3.5. Surface Morphology

**Figure 1:**
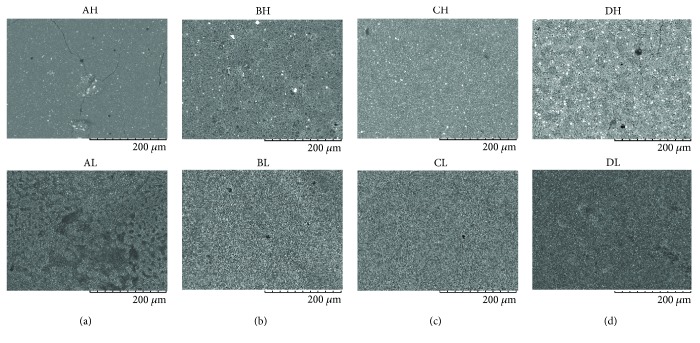
Immediate scanning electron microscopy images (400x) of Hydro C (H) and Life (L) cements with (a) no treatment; (b) phosphoric acid conditioning and rinsing; (c) rinsing only; and (d) polyacrylic acid conditioning and rinsing.

**Figure 2:**
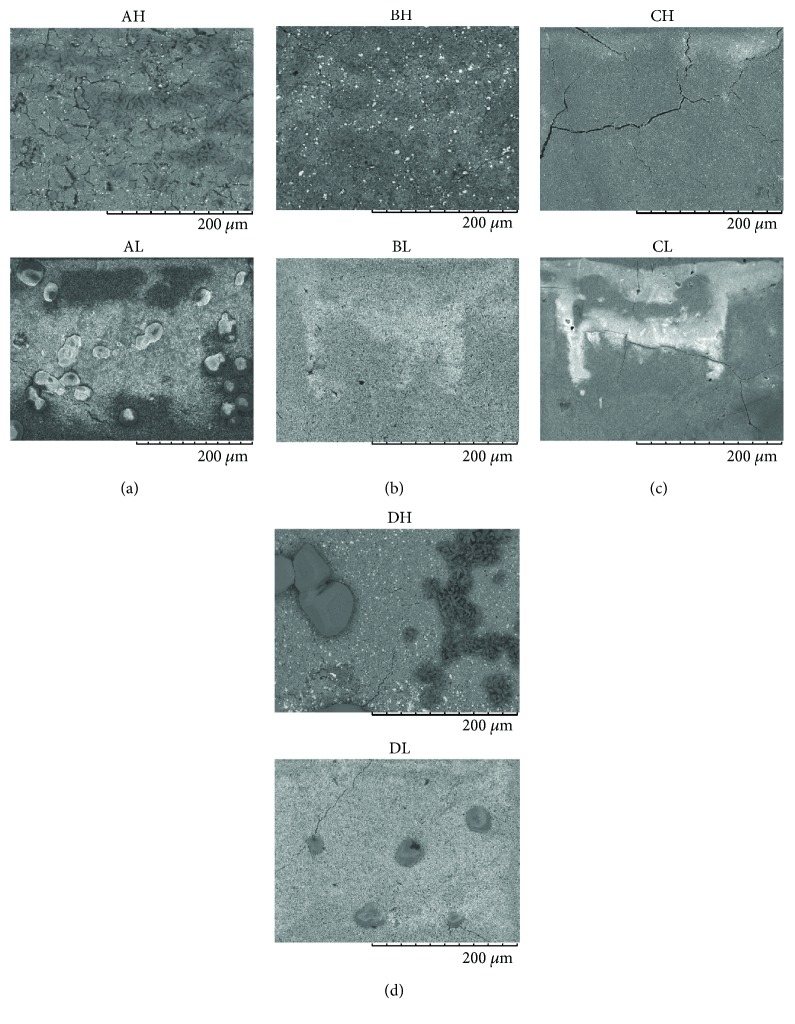
Scanning electron microscopy images (400x) of Hydro C (H) and Life (L) cements after storing in water for 7 days with (a) no treatment; (b) phosphoric acid conditioning and rinsing; (c) rinsing only; and (d) polyacrylic acid conditioning and rinsing.

For the immediate analysis, the control group showed a smoother surface for HyC (AH) compared with LF (AL), despite the presence of small fissures (Figure 1). For the analysis after 7 days, structures similar to mineral components and associated with deeper fissures increased significantly in the control group (Figure 2). In the PHO group, the analysis after 7 days showed no significant difference compared with the immediate analysis for both cements; a homogenous granular appearance was maintained (Figures 1 and 2), differing only in a heterogeneous appearance in the control group. For LF, the PHO, POL, and rinsing groups presented a similar surface appearance, differing only from the control group (Figures 1 and 2). The rinsing group showed a greater difference after the 7-day analysis, changing from a uniform (Figure 1) to a fissured surface (Figure 2) for both cements. For the POL group, mineral deposits were found after 7 days, very different from control group, which presented as heterogeneous and granular (Figure 2).

## 4. Discussion

The null hypothesis tested in the study that polyacrylic and phosphoric acids would have no influence on the physical and mechanical properties of Hydro C and Life calcium hydroxide cements was rejected. Contact with the acids affected water sorption, solubility, roughness, release of hydroxyl ions (pH), and surface morphology of calcium hydroxide cements.

One of the most important reasons for the use of calcium hydroxide cement as a liner is that alkaline pH is maintained, even after complete curing, as a result of continuous diffusion of hydroxyl ions when in contact with dentin fluid [14]. In this study, the release of hydroxyl ions (pH) from the distilled water in which the samples were immersed for 7 days was evaluated to assess if the alkalinity potential of the material was affected by exposure strategies. All the calcium hydroxide samples were able to alkalize distilled water, except for those exposed to PHO, which were statistically different compared with the others for both LF and HyC cements. For groups exposed to PHO, the pH remained virtually the same as that of the immersion water (pH 6.46), with HyC presenting the lowest value, significantly different from LF. That result can be explained by associating the solubility of calcium hydroxide materials exposed to phosphoric acid with release of ions. It has been reported considerable resistance to solubility for this type of acid, which can explain the low release of hydroxyl ions, maintaining the pH equal to that of distilled water used and corroborating the solubility results found in the present study [15]. This low potential for alkalization exacerbated in the groups treated with PHO suggests that the biological and therapeutic effects of these cements may be reduced when touched lightly by PHO. Although both LF and HyC cements increased the pH of the immersion water, both demonstrated a low alkalizing ability, with an average pH around 7.2. This result cannot be compared with others, because most previous studies did not assess the effect of PHO on the surface of cements regarding these properties [2, 16]. Therefore, solubility and release of hydroxyl ions are related properties, as shown by the results presented here.

With regard to water sorption, HyC cement had higher mean than LF when in contact with POL. That can be explained by the association between the type of conditioning agent and the basic composition of each cement used in the experiment. A previous study [17] has shown that in the presence of resinous particles, cements such as LF appear to have greater resistance to solubility and water sorption compared with conventional cement such as HyC. That result can be also related to the results of the solubility and release of hydroxyl ion tests in our study.

With regard to surface roughness, the cements behaved differently. The surface roughness of HyC increased with PHO, whereas LF showed a reduction in surface roughness, becoming superficially smoother after PHO and POL. Comparing both cements, only the groups treated with POL were statistically similar. This result may be explained by the different chemical composition of the cements as well as selectivity of the action of the acids used. As plasticizers are the main components responsible for controlling the disintegration of the material, this explains why the results differ; HyC contains ethyl toluene sulfonamide and LF contains butyl benzene sulfonamide. A previous study [15] using cements with both plasticizing components showed the same results regarding surface integrity, corroborating our results. Thus, the resinous phase of butyl benzene sulfonamide may be less stable than that of ethyl toluene sulfonamide regarding acid etching. Therefore, for HyC cement, the selectivity of the actions of the acids generates some loss of material, resulting in superficial peaks and valleys. Furthermore, LF cement presents considerable resistance to PHO penetration [18], explaining its smoother surface. Therefore, with greater loss of material caused by plasticizing, surface roughness was statistically higher for HyC after PHO and lower for LF.

In the present study, LF and HyC presented a statistically significant difference in relation to release of hydroxyl ions only for the PHO conditioning agent, with LF showing higher means. However, PHO caused lower surface roughness on LF compared with HyC. Therefore, it is possible that the dissolution caused by this acid was responsible for greater release of hydroxyl ions, but mostly reaching the peaks rather than the valleys. Thus, the smoother surface of LF cement after PHO conditioning acid observed immediately and after 7 days corroborates the results for both surface roughness and release of hydroxyl ions.

The immediate SEM analysis of LF revealed that this cement presents a thin granular and uniform surface [16], which corroborates the higher surface roughness found in the control group, especially when compared with HyC. Small fissures were found in the SEM analysis and they were aggravated after 7 days of storage. That may be the result of loss of some minerals with time, also proposed by Gandolfi et al. [12] The samples in the rinsing group also showed similar surface roughness to the samples in the control group; therefore, it is evident that the action of the acids was responsible for the surface alterations in this cement, leading to loss of constituents, reduction in the peaks and valleys, and thereby a smoother surface.

In the analysis of the surfaces after 7 days of storage, despite the fact that PHO with HyC resulted in a homogeneous granular aspect, the mean surface roughness was higher. Therefore, filler particles on the surface of the specimens found in the other groups did not lead to greater surface roughness, shown by isolated peaks and valleys in the morphological view. However, major mineral deposits found in the control, rinsing, and POL groups for LF cement on SEM corroborate the results of the roughness test, affirming this higher mean compared with HyC, because these particular deposits of precipitated minerals were not prevalent on the microscopic analysis.

In this study, the exposure strategies did not result in a statistically significant difference in resistance to impact when analyzing one cement. However, when comparing both cements, the results were statistically similar, except for the samples treated with POL. In this case, the values for impact strength were high for HyC than for LF. Some reduction in compressive strength may be attributed to dissolution or adsorption of water during storage [17]. In this study, LF had statistically significant differences from HyC in relation to dissolution of hydroxyl ions, and therefore this would not be a possible cause for its reduced resistance. In a previous study, it was observed that LF showed low solubility in PHO (60 s), high solubility in water (24 h), and a compressive resistance twice as high as other cements, including Dycal [19], which is in contrast to the above statement. Thus, it can be suggested that exposure of LF to POL resulted in some loss of major constituents affecting impact resistance.

In order to see if there is a gradient of microstructure of both cements, further investigations can be performed evaluating a cross section of samples. Indeed, clinical studies on protocols for indirect pulp capping are necessary, and the results obtained in laboratory studies must be carefully applied to clinical conditions, because calcium hydroxide-based cements are significantly more soluble in distilled water than in simulated dentin fluid or saliva [17]. Furthermore, in an in vivo situation, there is a continuous exchange of fluids from dental tissue to material's surface, which rapidly dilutes the effect of the pH of the calcium hydroxide [13, 20].

## 5. Conclusion

The calcium hydroxide cements tested in this study showed considerable changes in their physical and mechanical properties, such as sorption, solubility, release of hydroxyl ions (pH), surface roughness, and surface morphology, when in contact with polyacrylic and phosphoric acids. Water sorption of HyC and LF cements was affected after contact with PHO and POL, respectively. The solubility of both cements was affected only by contact with POL. The impact resistance of both materials was not affected by exposure to the acids. The release of hydroxyl ions from both cements was affected by contact with PHO. Contact with both acids affected the surface roughness of LF cement; only PHO affected the surface roughness of HyC cement.

## Figures and Tables

**Table 1:** Materials used in this study.

Materials and manufacturer	Composition	Lot number
Hydro C (DENTSPLY Caulk, Milford, DE, USA)	Catalyst: calcium hydroxide, zinc oxide, ethyl toluene sulfonamide, zinc stearate, and mineral dyes	064969H
	Base: ester glycol salicylate, barium sulfate, titanium dioxide, silica, and mineral dyes	
Kerr Life (Kerr, Karlsruhe, Germany)	Catalyst: bisacrylic and trisacrylic resin, methyl salicylate	4-1325
	Base: calcium oxide and zinc oxide	
Riva conditioner (SDI, Bayswater, Australia)	25–30% polyacrylic acid, balancing ingredients	140355
Super etch (SDI, Bayswater, Australia)	37% phosphoric acid, balancing ingredients	130694Z

**Table 2:** Means (standard deviation) of water sorption (*μ*g/mm^3^) according to the type of material and treatment.

Material	Conditioning agent			
	No treatment	Rinsing only	Polyacrylic acid and rinsing	Phosphoric acid and rinsing
Hydro C	2.6 (0.7)^aA^	3.0 (0.7)^aA^	3.7 (0.7)^aA^	2.9 (0.8)^aA^
Life	2.9 (1.0)^aAB^	2.1 (0.7)^aAB^	1.9 (0.6)^bB^	3.4 (1.2)^aA^

Different capital letters represent statistically significant differences between surface treatments of the same material (*P* < 0.05). Different lower case letters represent statistically significant differences between materials for the same surface treatment (*P* < 0.05).

**Table 3:** Means (standard deviation) of solubility (*μ*g/mm^3^) according to the type of material and treatment.

Material	Conditioning agent			
	No treatment	Rinsing only	Polyacrylic acid and rinsing	Phosphoric acid and rinsing
Hydro C	1.7 (0.7)^aAB^	2.2 (0.7)^aAB^	2.4 (0.7)^aA^	1.6 (0.8)^aB^
Life	2.0 (1.0)^aB^	2.0 (0.7)^aB^	2.3 (0.6)^aA^	1.8 (1.2)^aB^

Different capital letters represent statistically significant differences between surface treatments of the same material (*P* < 0.05). Different lower case letters represent statistically significant differences between materials for the same surface treatment (*P* < 0.05).

**Table 4:** Means (standard deviation) of surface roughness in Ra (*μ*m) according to the type of material and treatment.

Material	Conditioning agent			
	No treatment	Rinsing only	Polyacrylic acid and rinsing	Phosphoric acid and rinsing
Hydro C	0.50 (0.25)^bB^	0.26 (0.03)^bB^	0.52 (0.22)^aB^	1.82 (0.77)^aA^
Life	3.19 (0.15)^aA^	3.56 (0.32)^aA^	0.64 (0.23)^aB^	0.48 (0.08)^bB^

Different capital letters represent statistically significant differences between surface treatments of the same material (*P* < 0.05). Different lower case letters represent statistically significant differences between materials for the same surface treatment (*P* < 0.05).

**Table 5:** Means (standard deviation) of impact resistance in newtons (N) according to the type of material and treatment.

Material	Conditioning agent			
	No treatment	Rinsing only	Polyacrylic acid and rinsing	Phosphoric acid and rinsing
Hydro C	31.0 (9.5)^aA^	35.9 (7.6)^aA^	41.7 (10.1)^aA^	31.4 (8.9)^aA^
Life	31.9 (6.3)^aA^	33.1 (3.5)^aA^	26.9 (3.0)^bA^	28.4 (3.7)^aA^

Different capital letters represent statistically significant differences between surface treatments of the same material (*P* < 0.05). Different lower case letters represent statistically significant differences between materials for the same surface treatment (*P* < 0.05).

**Table 6:** Means (standard deviation) of pH according to the type of material and treatment.

Material	Conditioning agent			
	No treatment	Rinsing only	Polyacrylic acid and rinsing	Phosphoric acid and rinsing
Hydro C	7.2 (0.1)^aA^	7.2 (0.0)^aA^	7.2 (0.0)^aA^	6.6 (0.3)^bB^
Life	7.2 (0.0)^aA^	7.1 (0.0)^aA^	7.1 (0.0)^aA^	6.8 (0.0)^aB^

Different capital letters represent statistically significant differences between surface treatments of the same material (*P* < 0.05). Different lower case letters represent statistically significant differences between materials for the same surface treatment (*P* < 0.05).

## Data Availability

The data used to support the findings of this study are available from the corresponding author upon request.
